# No evidence of systematic pre-emptive loggings after notifying landowners of their lands’ conservation potential

**DOI:** 10.1007/s13280-020-01354-4

**Published:** 2020-06-23

**Authors:** Eini Nieminen, Kalle Salovaara, Panu Halme, Janne Sakari Kotiaho

**Affiliations:** 1 Department of Biological and Environmental Science, University of Jyväskylä, P.O. Box 35, 40014 Jyväskylä, Finland; 2 School of Resource Wisdom, University of Jyväskylä, P.O. Box 35, 40014 Jyväskylä, Finland

**Keywords:** Environmental policy, Forest conservation, Mire conservation, Panic clearing, Peatland, Private protected area

## Abstract

Landowners can intentionally impair biodiversity values occurring on their land to pre-empt biodiversity protection. This often leads to significant negative effects on biodiversity. We studied whether landowners in Finland engaged in pre-emptive loggings after they were notified that their wooded mires are candidate sites for a mire protection program. After the notification, harvesting rates of the candidate wooded mires were significantly lower compared to harvesting rates of similar but non-candidate wooded mires. Annual and monthly harvesting rates indicated that notifying landowners of the conservation potential did not launch systematic pre-emptive logging behavior. Nevertheless, part of the candidate wooded mires were logged, so some landowners place more weight on other values than the biodiversity ones. Pre-emptive behavior has been observed in other studies suggesting that many country- or system-specific factors such as cultural background or level of compensation can affect landowners’ behavior.

**Electronic supplementary material:**

The online version of this article (10.1007/s13280-020-01354-4) contains supplementary material, which is available to authorized users.

## Introduction

Anthropogenic activity often degrades habitats resulting in reduction or even eradication of species’ populations (Newbold et al. 2015). Therefore, restrictions in land use practices are an inevitable consequence of biodiversity protection. Land use restrictions are known to cause conflicts especially when conservation is based on command-and-control approaches such as the Endangered Species Act in the USA or the conservation program Natura 2000 in Europe. Both have been shaped with contradictions followed by for e.g., a lack of communication, information sharing, stakeholder involvement, and justice (e.g., Paavola 2003; Grodzinska-Jurczak and Cent 2011; Blicharska et al. 2016; Olive 2016). Landowners of areas hosting endangered species or habitats can have negative attitudes towards conservation actions for several reasons. For instance, land use restrictions to protect biodiversity can be considered as insulting property rights, being unfair actions, or causing economic harm (e.g., Jackson-Smith et al. 2005; Kabii and Horwitz 2006; Kamal et al. 2015; Blicharska et al. 2016; Olive 2016; Jokinen et al. 2018).

Command-and-control approaches can generate perverse incentives to intentionally destroy or damage species or habitats. Such behavior is here referred to as pre-emptive behavior. Landowners can manage their lands in ways that harm threatened species directly (Brook et al. 2003; Jokinen et al. 2018). Occurrences of threatened species can also lead to shortened rotation times of forest loggings or to an increased probability of forests becoming logged on nearby sites, thereby preventing the species from dispersing into new areas (Lueck and Michael 2003; Zhang 2004). Net reduction of forest area caused by pre-emptive behavior can also outcompete attempts to halt deforestation (Simmons et al. 2018a).

Net effects of command-and-control approaches on biodiversity have both positive and negative outcomes. The Endangered Species Act in the USA seems to protect species from extinctions and increases the likelihood of species’ status to improve (Schwartz 2008), at least if species’ listings to the Act are combined with sufficient species-specific funding (Ferraro et al. 2007; Gibbs and Currie 2012). Still, negative impacts of pre-emptive behavior on single species may be significant (Brook et al. 2003; Lueck and Michael 2003). In Australia, strict clearing bans based on the Vegetation Management Act have increased forest cover on some regions and forest types, but later changes and uncertainties in the implementation of the Act have caused pre-emptive deforestation and other perverse effects leading to a net loss of remnant forest patches (Simmons et al. 2018b).

While evidence about pre-emptive behavior comes mainly from the USA (e.g., Brook et al. 2003; Lueck and Michael 2003; Zhang 2004) and Australia (Simmons et al. 2018a, b), the topic is debated also in many other parts of the world. Increasingly more land is converted to human use and, consequently, the loss of biodiversity continues (Pereira et al. 2010; Lambin and Meyfroidt 2011; IPBES 2019). The role of private lands in biodiversity protection is increasing as these host significant proportions of distributions of many endangered species and habitats (e.g., Knight 1999; Norton 2000). It is likely that landowners in different countries may respond differently to the risk of land use restrictions caused by conservation actions. Such difference may arise due to e.g., previous environmental administrative practices or politics (Paloniemi and Vilja 2009), a cultural-specific relationship with nature and land (Silvasti 2003), or compensation practices (Byl 2019).

Establishing the European Union’s Natura 2000 conservation network caused heavy opposition by local people throughout Europe (Alphandéry and Fortier 2001; Hiedanpää 2002; Paavola 2003; Grodzinska-Jurczak and Cent 2011). The opposition initiated a development towards voluntary-based conservation approaches during the 21st century (Keulartz 2009). Since then, voluntary nature protection has been a predominant tool in forest conservation in Finland, but for other habitat types similar administrative tools are still lacking (Council of State 2014; Paloniemi and Vilja 2009). In 2012, the Complementary Mire Protection Program (hereafter the CMPP) aiming to extend the national mire conservation network was politically agreed on to be based on the Nature Conservation Act (1096/1996) which allowed the CMPP to be implemented by means of land expropriations, including a full financial compensation or land exchange to landowners (Council of State 2012). The CMPP was later converted to a voluntary program, rejecting the option of expropriations (Salomaa et al. 2018; Nieminen et al. in review). However, before the rejection, landowners of mires with conservation potential were notified about the CMPP. The notification could have provoked owners of wooded mires to conduct logging in order to avoid their lands from being protected. Claims and anecdotes of such actions exist in social media sources like Twitter and forums of forestry magazines.

The aim of this paper is to determine if notifying landowners of their lands’ conservation potential led to pre-emptive loggings on Finnish wooded mires. We analyzed whether harvesting rates of wooded mires chosen as candidate sites for the CMPP differed from harvesting rates of all other similar wooded mires in Finland that were not candidates for the CMPP. We also compared annual and monthly harvesting rates of mires with and without the candidate status to see whether events linked to the CMPP, such as notifying landowners of the conservation potential, caused sudden increase in the harvesting rates of the candidate wooded mires. To our knowledge, this is the first quantitative, nationwide analysis on pre-emptive behavior in Europe.

## Materials and methods

### Study case

In its preparation phase, the CMPP covered 327 300 ha of unprotected candidate mires considered for protection (Alanen and Aapala 2015; Kareksela et al. 2020). The aim was to protect about 100 000 ha of the ecologically most important mires to complement the existing mire protection network in Finland.

Originally in August 2012, the CMPP was politically agreed to be based on the Nature Conservation Act which enables land expropriations for conservation purposes (Council of State 2012). Practically, owners of the lands chosen for protection would have been allowed to decide whether to keep the ownership of the land, resulting in a private conservation area, or to sell it to the government. In both cases, landowners would have been compensated by being paid a market price for their land, or by exchanging their land for an equivalent parcel of the government’s land elsewhere, depending on landowner’s will.

The public briefing of the CMPP started in the beginning of 2013 by announcements in newspapers, a poll in a government-operated citizen portal in the internet, and hearings of stakeholder representatives. In May–July 2013, landowners of candidate mires received personal information letters notifying about field inventories that were made for the preparation of the CMPP during the summer 2013. In the autumn 2014, just before its implementation, the CMPP was revised to a voluntary program and the option of land expropriations was rejected due to political turmoil (Salomaa et al. 2018). This changed the CMPP’s preparation and implementation remarkably. At that time, the CMPP provoked plenty of public deliberation. In the autumn 2015, 117 000 ha of the most ecologically valuable mires were proposed to be protected, but proper administrative tools to implement their protection did not exist. Further political changes, such as cuts of conservation resources, left all but the government-owned proposed mires without protection. Afterwards, the CMPP has regularly appeared in the media and is mentioned also in the current Finnish Government Program (Anonymous 2019). In the current conservation policy, however, there are no signs of land expropriations being re-allowed in the CMPP.

Characteristics of wooded mires supporting their typical biodiversity features are connected to their tree stand and intact hydrological and microclimatic conditions (Laine et al. 1995; Maanavilja et al. 2014). Therefore, landowners resisting protection may easily impair conservation values of wooded mires with pre-emptive loggings. Landowners had, and still have, a possibility to log their wooded mires, since commercial forestry is legal on most of the mires considered to be included in the CMPP.

### Study design

To ensure long-term effectiveness of conservation, candidate mires were planned to form hydrological entities (Aapala and Alanen 2015; Kareksela et al. 2020). Therefore, the candidate mires also included small patches of forests on mineral soils. We outlined the study to include only wooded habitat types occurring on peatlands in the boreal zone, i.e., spruce and pine mires. In Finland, both are commonly in a forestry use. For photographs of typical boreal spruce and pine mires, see Fig. S1.

We composed four groups of mires. The experimental group was composed of wooded mires with the candidate status and the control group of wooded mires without the status. Ideally, the study design would have included candidate mires of both informed and uninformed landowners, but in our real-world case all owners of candidate mires had been informed about their mires’ conservation potential. Therefore, our study design was the best possible way to address the research questions.

Experimental and control groups were divided into spruce mires and pine mires. We analyzed harvesting rates during 5 years after the initial notification (2013–2017) and, additionally, stratified the data into annual and monthly harvesting rates. Concerning the monthly harvesting rates, we were especially interested in May–July 2013 when landowners received a notification of their lands being potential mires for protection, and October–November 2014 when the CMPP was revised to be a voluntary one. As a response variable for the overall harvesting rates over the 5 years and the annual harvesting rates, we calculated the area (hectares) that was logged and unlogged within each of the groups. As a response variable for the monthly harvesting rates, we utilized the number of submitted forest use notifications. We used the number rather than the hectares covered by the notifications because the sample sizes for monthly logged sites were small. In such a case the hectares might have masked the effect because an area covered by a notification varies, but a notification itself always reflects a landowner’s behavior independent from the area. For figures showing the differences in the harvesting rates created according to logged area or submitted forest use notifications, see Appendix S1.

Candidate mires were mostly in a natural state or close to it. If they had been highly modified or degraded by human activity, they would not have been chosen as potential sites to the CMPP. Due to the desire to protect candidate mires as hydrological entities, some of them enclosed small degraded parts which were planned to be restored after protection. However, the average age and timber volume of candidate wooded mires likely represent those of older forests. To make the experimental and the control groups to be equivalent, we included to the analyses only those candidate and non-candidate wooded mires that were of the two most mature forest development class (advanced thinning stands and mature stands, see Appendix S2). We also calculated average diameters of trees in logged candidate and non-candidate mires and found that they did not differ remarkably, indicating that their timber quality was similar (Table S1).

We set the period of the study to be January 2013–December 2017. Since the CMPP was publicly briefed from January 2013 onwards, it was not reasonable to study harvesting rates earlier. If candidate wooded mires had been logged earlier, they would not have been selected as the candidates in the first place.

In Finland, the Forest Act (1093/1996) obliges forest owners to make a notification of forest use before logging. Practically, all loggings are executed after submitting a notification, since an industrial agent such as a timber buyer or a logging planner commonly makes the notification. Illegal loggings are very rare which is verified by a well-working law enforcement (Finnish Forest Centre 2018). Therefore, we applied notifications as surrogates for the loggings.

### Data

For the analyses, we compiled eight different spatial data: unlogged and logged spruce and pine mires with the candidate status, and unlogged and logged spruce and pine mires without the status (Table 1).

**Table 1:** Sample sizes of the final processed data

Candidate status	Habitat type	Logging status	Hectares	No. of notifications
Mires with the candidate status	Spruce mires	Unlogged	2198	na
		Logged	183	235
		Total	2381	235
	Pine mires	Unlogged	6661	na
		Logged	981	700
		Total	7642	700
Mires without the candidate status	Spruce mires	Unlogged	357 415	na
		Logged	78 196	54 314
		Total	435 611	54 314
	Pine mires	Unlogged	599 896	na
		Logged	136 390	61 473
		Total	736 286	61 473

To compile data of all wooded mires in Finland, we utilized publicly available spatial forest resource data which include detailed information of Finnish forests (https://urly.fi/1jgz). It covers the majority of privately owned forest land but mostly it does not include government- and municipalities-owned lands (Appendix S3). However, as the majority of forest land in Finland is privately owned (Official Statistics of Finland 2011–2016), the data coverage can be considered representative. To restrict the data of all wooded mires to cover only advanced thinning or mature spruce and pine mire stands, we outlined the forest resource data according to a habitat type and a forest development class.

To compile data of all logged wooded mires in Finland, we utilized publicly available spatial data of forest use notifications which include information of logged forest stands (https://urly.fi/1jgF) (Appendix S3). It served as the source for all the wooded mire stands that were advanced thinning or mature ones and logged in 2013–2017. However, many of the notifications lacked information of the habitat type since it is not an obligatory field in the notification. To complete the habitat type information, we joined the notification data with the abovementioned data of all wooded mires and set the latter to act as a primary source for the habitat type. However, the data of all wooded mires did not cover all stands in the forest use notification data. We checked whether the notifications on these stands included the habitat type information, and if they did, it was used as the source for the habitat type. If the habitat type information was not available in either of the data, we excluded the stand in question from the analysis.

We detached the data of all logged wooded mires from the data of all wooded mires after which we had four data: unlogged and logged spruce and pine mires covering whole Finland.

The final eight data of unlogged and logged non-candidate and candidate spruce and pine mires were compiled by detaching the candidate spruce and pine mires from the abovementioned data of all unlogged and logged wooded mires. We made this by means of a separate data that covered locations of the CMPP’s candidate sites (Alanen and Aapala 2015; Kareksela et al. 2020).

### Final data processing

Assembling the datasets caused multiple fragment stands that were too small to be real forest stands. We analyzed the size distributions of the forest stand fragments separately for all eight datasets and estimated that excluding stands ≤ 0.14 ha would reduce the number of artificial stands without eliminating many of the real small stands (Appendix S4). It is likely that we did not succeed in excluding all the artificial stands and likewise, we possibly excluded some of the existing small stands. However, we found no reason to expect any bias in the data caused by the exclusion and, therefore, consider the data to be reliable. All the data were processed with ArcMap 10.

### Statistical analysis

We analyzed the harvesting rates per 5 years on mires with and without the candidate status and separately for pine and spruce mires against randomized harvesting rate distributions. To create the distributions, we set the total logged hectares of all pine and spruce mires to randomly locate on the whole area of the respective habitat. Randomization was performed with RStudio version 1.1.456 and replicated 1000 times. Replicates were compiled into a distribution describing how large proportion of logged hectares would randomly locate on the candidate mires of each habitat type. For the R-script, see Appendix S5.

## Results

**Fig. 1:**
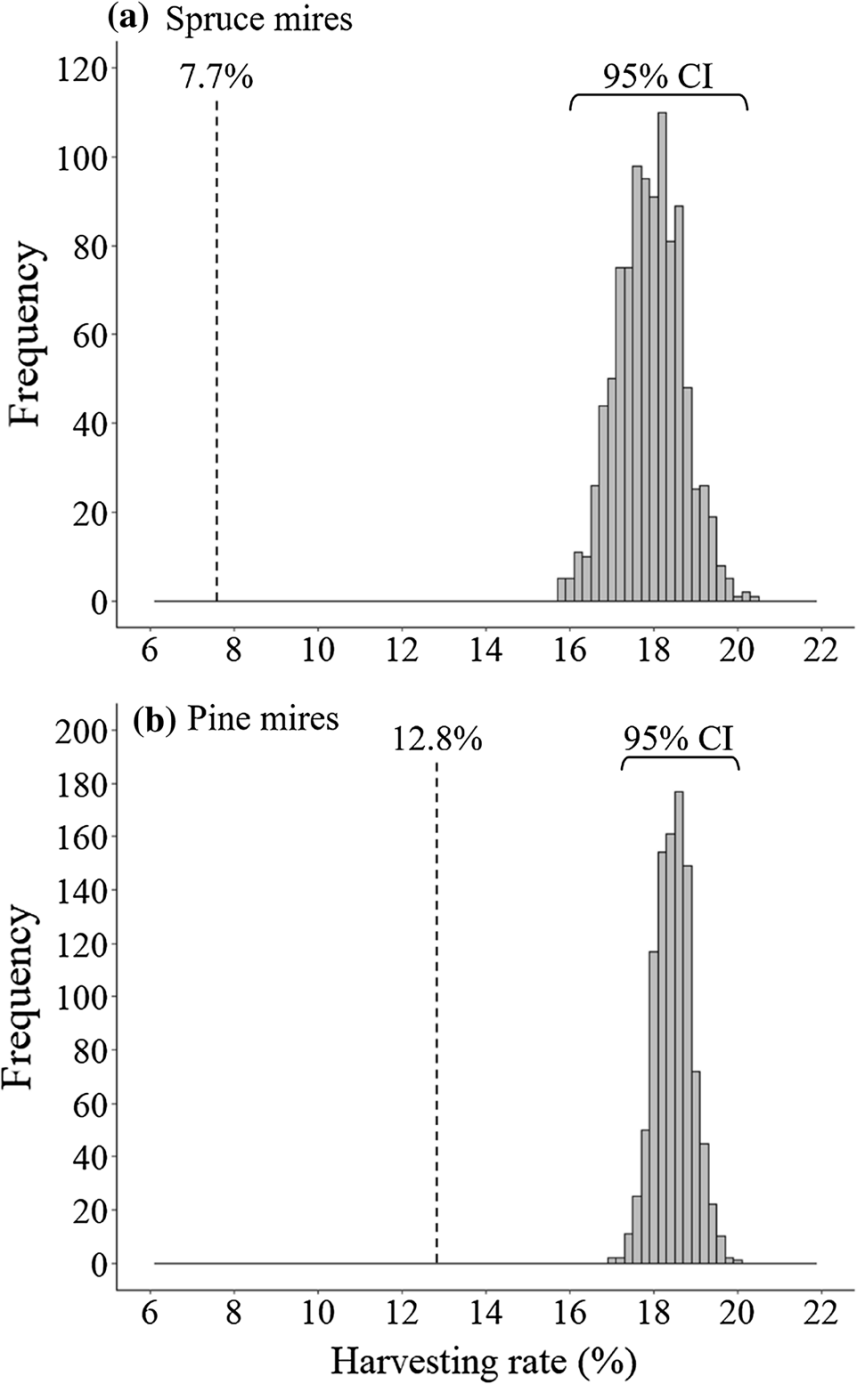
Gray bars show how large proportion of logged hectares would randomly locate on candidate **a** spruce mires and **b** pine mires, when randomization is replicated 1000 times. Actual harvesting rates per 5 years on candidate mires are marked with dashed vertical lines

7.7% (183 ha) of spruce mires and 12.8% (981 ha) of pine mires with the candidate status were logged based on hectares covered with submitted forest use notifications. Respective numbers for spruce mires without the candidate status were 18.0% (78 916 ha) and for pine mires 18.5% (136 390 ha). Therefore, the candidate mires were logged significantly less than the non-candidate ones (Fig. 1). For a map describing locations of all candidate mires and logged and unlogged wooded candidates, see Fig. S2.

**Fig. 2:**
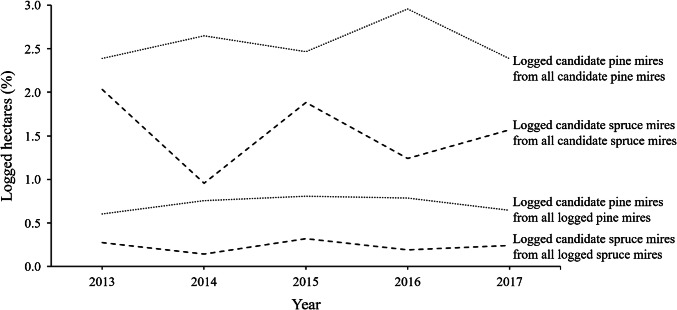
Habitat-specific annual harvesting rates on candidate wooded mires calculated from all candidate wooded mires and from all logged wooded mires. Calculations of logged areas are based on hectares covered by submitted forest use notifications. Dashed lines represent spruce mires and dotted lines pine mires

Notifying landowners of their mires’ conservation potential in May–July 2013 or revising the CMPP to a voluntary one in October–November 2014 did not produce harvesting peaks according to the annual harvesting rates that were calculated based on hectares covered with submitted forest use notifications (Fig. 2). In relation to the area of all candidate spruce or pine mires, the average annual harvesting rates on them were 1.54% and 2.57%, respectively. On candidate spruce mires, the harvesting rate was highest in 2013 (2.03% of all candidate spruce mires logged), whereas on pine mires, it was highest in 2016 (2.96% of all candidate pine mires logged). Candidate spruce mires were logged least in 2014 (0.96%) and pine mires in 2013 and 2017 (2.39% in both years). In relation to the area of all logged wooded mires, the logged area of candidate wooded mires was very low: on candidate spruce mires it varied between 0.14 and 0.32% and on candidate pine mires between 0.60 and 0.81%.

**Fig. 3:**
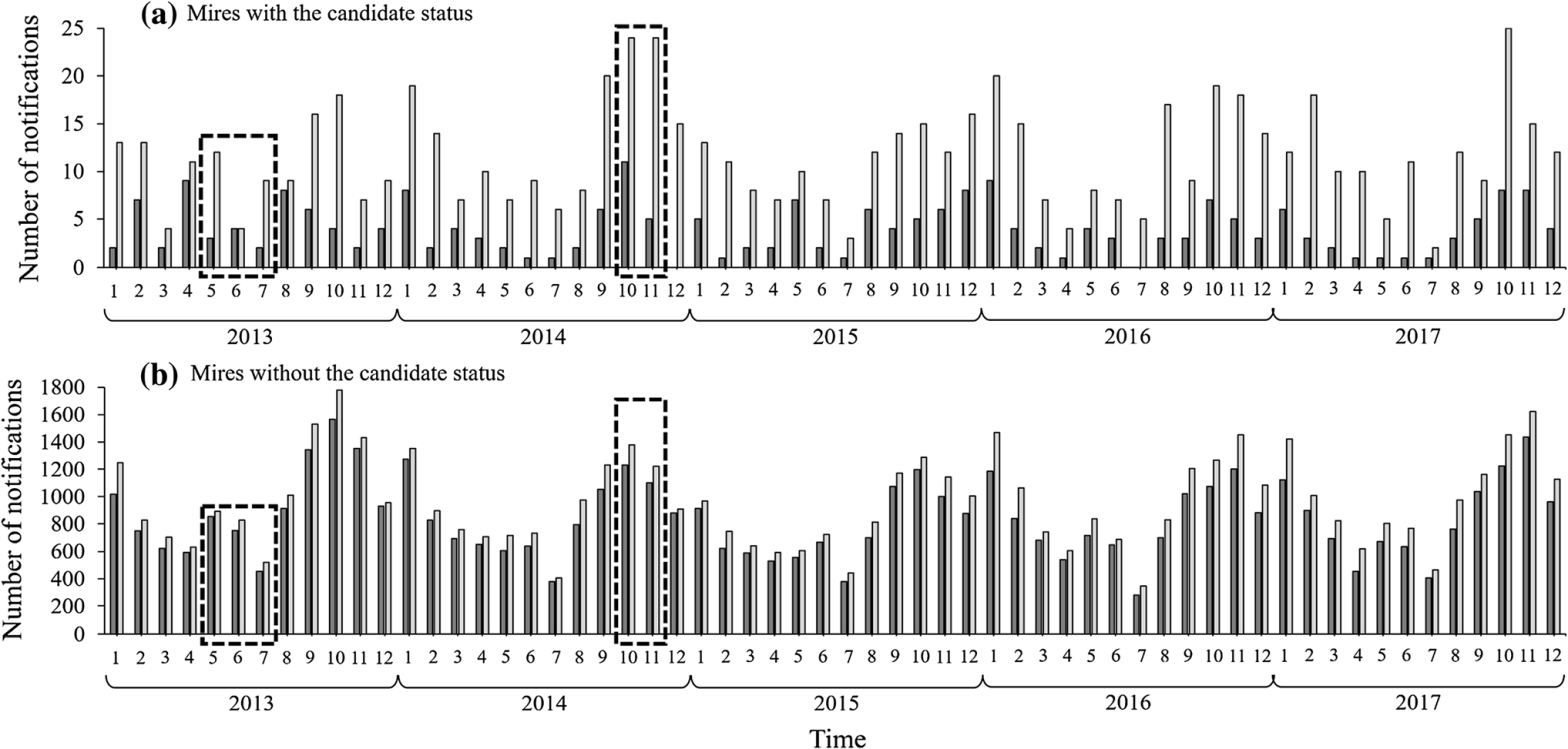
Numbers of forest use notifications submitted per month in 2013–2017. Dark gray bars represent spruce mires and light gray bars pine mires. **a** Mires with the candidate status. **b** Mires without the candidate status. The boxes with dashed lines represent the months when we expected the number of notifications to rise due to the certain events concerning the CMPP

Within the years, both candidate and non-candidate mires had seasonal variation in numbers of submitted forest use notifications (Fig. 3). Notifications were submitted more in autumn and winter, and less in spring and summer. On candidate spruce mires, the highest numbers of notifications during the study period were submitted in October 2014 (11 notifications), in April 2013, and in January 2016 (9 notifications during both). Respective months and years for candidate pine mires were October 2017 (25 notifications), and October and November 2014 (24 notifications during both). Taking into account the seasonal variation, the numbers of submitted notifications did not peak in May–July 2013, when landowners were notified that their mires are candidates for the CMPP, nor in October–November 2014, when the option of land expropriations was rejected.

## Discussion

Our main finding was that notifying landowners of their mires’ conservation potential and the possibility of mires becoming included in the CMPP did not cause systematic pre-emptive loggings. Instead, candidate wooded mires were logged significantly less than mires that were not considered for protection. The result is different from previous studies. In the USA, landowners have intentionally damaged species and habitats by applying shorter rotation times of loggings (Lueck and Michael 2003; Zhang 2004) and by changing land management practices (Brook et al. 2003). In Australia, pre-emptive behavior has caused loss of remnant forests (Simmons et al. 2018b).

While our results are encouraging, the root causes of the differences between our results and those of the earlier studies deserve further discussion. It is likely that landowners’ behavior is shaped by the society they live in. Majority of Finns, regardless of their socioeconomic or demographic status, agree that protection of mire habitats and species is important (Tolvanen et al. 2013). This finding was supported also by a survey that was conducted to the citizen owners of the candidate mires during the preparation of the CMPP: almost half of the respondents had a positive attitude towards protection of their mires (Alanen and Aapala 2015). The rise of voluntary nature conservation following from Natura 2000 and other command-and-control approaches have likely helped to overcome previous biodiversity conflicts in Finland (Paloniemi and Vilja 2009), possibly making the public attitude receptive to new conservation initiatives. Furthermore, there is evidence that a fair conservation compensation decreases the likelihood of pre-emptive behavior and increases the likelihood of pro-conservation behavior (e.g., Langpap 2006; Ferraro et al. 2007; Byl 2019). According to Finnish legislation, landowners are eligible to a market price compensation of economic losses caused by land expropriations for biodiversity protection or for any other societally significant purposes. Hence, one reason for the lack of systematic pre-emptive behavior in our case may be that Finnish landowners perhaps do not feel their livelihood is seriously threatened by land use restrictions. In contrast, land use restrictions set e.g., by the Endangered Species Act in the USA are not compensated monetarily. Instead, if landowners pledge to certain conservation activities, they can be compensated e.g., by providing various assurances of not to set further restrictions on their land (Donahue 2005). Hence, principles of compensation are fundamentally different in Finland compared to the USA.

To determine if there were any obvious relationships between the CMPP’s events and temporal patterns of the logging activity, we inspected yearly and monthly harvesting rates. The yearly harvesting rates were rather constant, but the monthly harvesting rates varied seasonally. This is explained by weather conditions favoring timber harvesting in autumn and winter when the ground is frozen and holds up forest harvesters. In May–July 2013, landowners of candidate wooded mires were notified that their mires are potential sites for protection. There was no detectable change in the monthly harvesting rates on candidate wooded mires relative to non-candidate ones, indicating that notifying landowners did not cause an increase in the logging activity. Another event potentially increasing the harvesting rates on candidate wooded mires was the decision of changing the CMPP to a voluntary program and rejecting the option of land expropriations in October–November 2014. After this, some landowners could have thought they had an opportunity to harvest without a disapproval of neighbors or the society at large (Jackson-Smith et al. 2005). To some landowners, the coverage of the conflict could have served as a reminder of the CMPP’s preparation, or even as a support for defiance against biodiversity protection. However, there were again no obvious change in the monthly harvesting rates on candidate wooded mires relative to non-candidate ones.

In a comparative study like ours, there is always a possibility that some other factors than the ones being explored have had an impact on the response variable. In our case, the characteristics of tree stand on candidate wooded mires and their non-candidate counterparts were similar (Table S1), but candidate wooded mires had on average higher biodiversity values since they were chosen as potential protected areas. In order to host high biodiversity, candidate wooded mires could not have been largely exposed to former land use practices such as ditching or logging since mires’ typical biodiversity features are dependent on tree stand and intact hydrology (Laine et al. 1995; Maanavilja et al. 2014). At least two factors could explain why candidate wooded mires were less exposed to land use in the first place, possibly affecting also landowners’ responses to informing about their mires’ conservation potential. First, it is possible that candidate wooded mires could have been on average smaller sized than non-candidate ones. This is because in the era of heavy ditching campaign in the 1960s and 1970s (Vasander 2006), large mires having a high potential for wood production or peat mining were probably more likely ditched than smaller mires. Second, it might be that candidate wooded mires locate further away from roads than their non-candidate counterparts. If candidate wooded mires were on average smaller and/or more remote than non-candidate ones, they could have been silviculturally less attractive. In Finland, however, Forestry Management Associations often endeavor to centralize loggings to certain areas so that neighboring forest properties are logged at the same time, lowering the logistical costs of small-sized regeneration ready stands. This balances the effect of possibly smaller average size of candidate wooded mires on landowners’ willingness to log. Unfortunately, we were not able to calculate the areas of single candidate or non-candidate mires since their borders were lost due to the data processing (see Materials and Methods). Furthermore, over 99% of forest land in central Finland locates < 400 m from the nearest road (Viitala et al. 2004) and the government supports construction of new forest roads in the whole country (Temporary Act on the Financing of Sustainable Forestry 34/2015), so it is improbable that remoteness would have prevented landowners to log their candidate wooded mires. Even if there were some other reasons for the candidate mires’ low harvesting rates than landowners’ awareness of their lands’ conservation potential, the lack of obvious increases in the logging activity on candidate mires after notifying of their conservation value means that landowners did not engage in systematic pre-emptive loggings.

Despite the low harvesting rates, some of the candidate wooded mires were nevertheless logged in each study year. The rather constant yearly harvesting rates of candidate wooded mires, the seasonal variation in their harvesting rates imitating that of non-candidate wooded mires, and the lack of obvious logging peaks after notification letters implicate that instead of intentionally harming biodiversity values, there may have been some other reasons to log. Landowners may have simply followed their long-term logging plans that are often made in cooperation with local forestry specialists. Evidence shows that forestry-oriented landowners trust forestry specialists and prefer cooperating with them also in conservation issues rather than with environmental authorities (Paloniemi et al. 2006). Therefore, forestry-oriented landowners may have actively disregarded the information provided by the environmental authorities about their mires’ high conservation potential. Such behavior may be expected particularly if a landowners’ income is dependent on the actualized loggings. There is earlier evidence of Finnish forest owners intentionally taking actions to harm flying squirrel (*Pteromys volans*) (Jokinen et al. 2018), so it is also possible that some owners of candidate wooded mires executed intentional pre-emptive loggings.

Since some mire owners are conservation minded (Alanen and Aapala 2015), it might be possible that at some point, logging candidate wooded mires would reduce even without protection. However, random factors such as transferring land property to the next generation or sudden acute need of money may initiate logging of a biodiversity-rich but non-protected mire even if the current owner would have decided to set the mire aside by his/her own decision. Excluding biodiversity-rich areas from official protection is a potential threat for long-term persistence of biodiversity, since forestry in Finland is so intensive that majority of forest sites will be logged when they reach maturity (Natural Resources Institute Finland 2019). If loggings on candidate wooded mires continued with the observed average annual rate of 36.6 ha (1.54%) for spruce mires and 1962 ha (2.57%) for pine mires, it would take only 26 and 13 years to loose half of them, respectively. Additionally, the likelihood to reach an ecologically representative mire conservation network decreases as increasingly larger area of candidate wooded mires are exposed to loggings. Originally, from 327 300 ha of candidate mires, 117 000 ha were proposed for protection (Alanen and Aapala 2015; Kareksela et al. 2020), leaving 210 300 ha without a protection request. Revising the CMPP to a voluntary program enabled landowners to refuse protection, inevitably changing the combination of mires applicable for protection (Nieminen et al. in review). In this new situation, 210 300 ha of mires originally not proposed to the CMPP could serve as compensatory sites for the mires that would be left out from protection due to some landowners’ unwillingness to protect. Therefore, logging both candidate wooded mires included in and excluded from the most ecologically valuable ones is problematic.

## Conclusions

Avoiding land use regulations by intentionally harming certain species or habitats has been proved to be a true phenomenon in the USA and Australia. We made the first quantitative exploration of pre-emptive behavior in Europe by studying logging behavior of landowners in Finland after they were notified that their wooded mires are candidate sites for a program that aims to extend the national mire conservation network. Unlike previous studies, we did not find evidence of systematic pre-emptive behavior. It is likely that landowners’ responses to potential land use restrictions caused by biodiversity protection depend on the country- or region-specific administrative, political, and cultural circumstances such as previous experiences of biodiversity conservation or the compensation practices. It is also possible that silvicultural characteristics of wooded mires such as harvesting restricted mainly to periods of frozen ground, or on average lower value of peatland forests compared to mineral soil forests can affect landowners’ behavior so that the results could have been different if the study was focused on mineral soils. Therefore, determining the exact reasons for the low harvesting rates of candidate wooded mires and the lack of systematic pre-emptive behavior demands further research such as mapping of landowners’ attitudes, motives, and beliefs. Nevertheless, our results are encouraging in showing that informing landowners openly about their lands’ conservation potential does not categorically lead to pre-empting of conservation values on wooded mires.

## Electronic supplementary material

Below is the link to the electronic supplementary material.

Supplementary material 1 (PDF 1218 kb)
