# Supplementary material for: No evidence of systematic pre-emptive loggings after notifying landowners of their lands’ conservation potential
Source: Ambio. 2020 Jun 23;50(2):465–74. doi: 10.1007/s13280-020-01354-4 (PMC7782606; doi:10.1007/s13280-020-01354-4)
Supplement: Supplementary file 1 — Supplementary material 1 (PDF 1218 kb) [file 13280_2020_1354_MOESM1_ESM.pdf]

Ambio

Electronic Supplementary Material

This supplementary material has not been peer reviewed.

Title: **No evidence of systematic pre-emptive loggings after notifying landowners of their lands' conservation potential**

Figure S1

a)

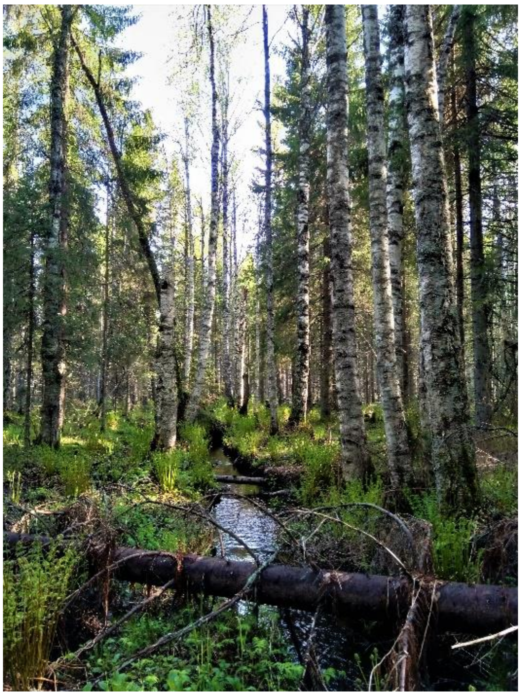

b)

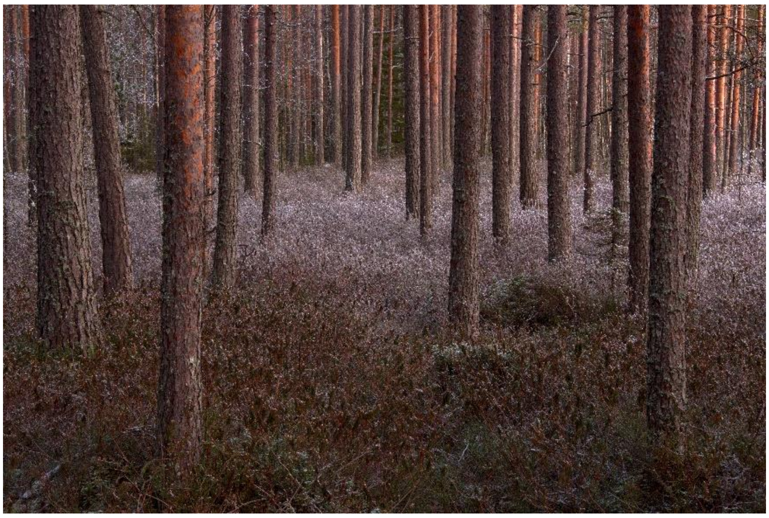

Figure S1. In Finland, boreal wooded mires are commonly in a forestry use. a) Boreal spruce mire. Spruce mires often have a large spruce-dominated tree stand, but also single deciduous trees or tree groups consisting of e.g. birch, alder, and aspen are typical. Photo taken by Eini Nieminen. b) Boreal pine mire. Pine mires have a pine-dominated tree stand which volume varies according to e.g. hydrological and nutrient-related factors. Photo taken by Petri Nevalainen.

Figure S2

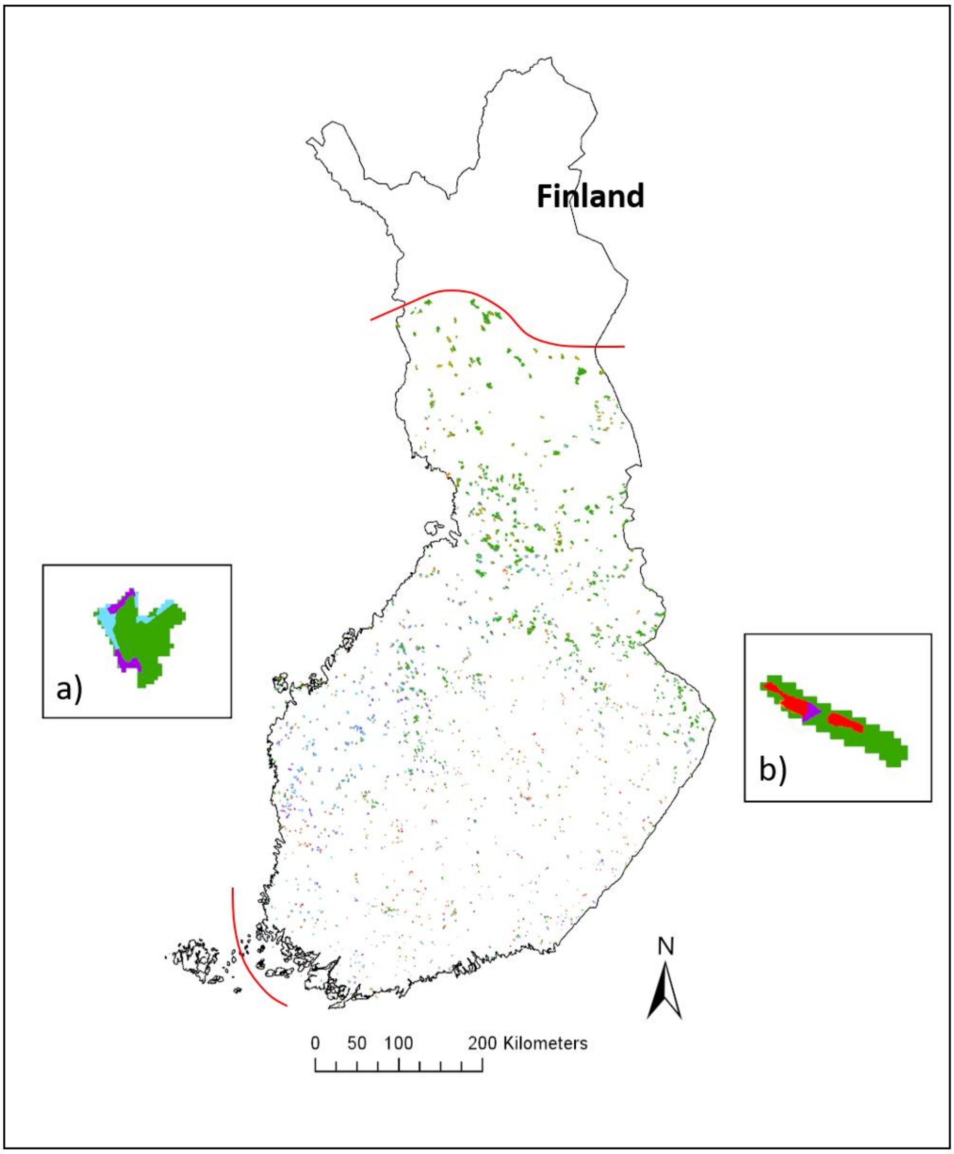

Figure S2. All candidate mires (green), unlogged candidate spruce mires (orange), logged candidate spruce mires (red), unlogged candidate pine mires (light blue), and logged candidate pine mires (purple). Red lines show the borders of the planning area in the CMPP. Two index maps (not in scale) show examples of executed loggings on candidate mires. a) The candidate mire locates in south-west Finland. Its area is 71 ha of which about 7 ha has been logged. b) The candidate mire locates in eastern Finland. Its area is 20 ha of which about 6 ha has been logged. To protect landowners' privacy, the exact locations of the candidate mires shown in the index maps are not shown.

Appendix S1

Comparison between area covered with forest use notifications and numbers of forest use notifications

As a response variable for monthly harvesting rates, we utilized numbers of submitted forest use notifications as with small sample sizes of candidate wooded mires, the number of notifications reflected landowners' behavior better than the area covered by notifications. To demonstrate this, we drew exemplary figures for spruce mires. Fig. S3b shows that e.g. in October 2014, the number of submitted notifications on candidate spruce mires was high compared to the area covered with notifications, whereas in February 2017, just few notifications were submitted, but the area covered with notifications was relatively high. On spruce mires without the candidate status, sample sizes were so high that there were not obvious difference between patterns drawn according to the number of notifications and the area covered by notifications (Fig. S3a).

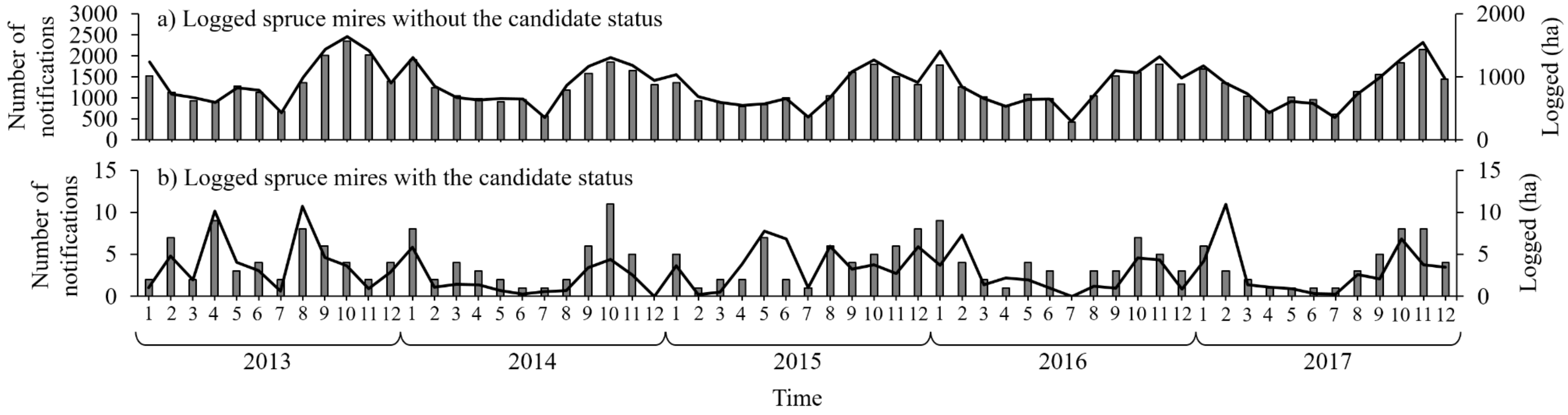

Figure S3. Comparison of harvesting rates drawn according to the number of submitted forest use notifications (bars) and the area covered with notifications (black lines). a) Logged spruce mires without the candidate status. b) Logged spruce mires with the candidate status.

Appendix S2

Forest development classes

We included to the analyses only those candidate and non-candidate wooded mires that were of the two most mature forest development class, i.e. advanced thinning stands and mature stands. The definition of advanced thinning stand is following: *"A forest stand where the average diameter on a breast-high is more than 16 cm, but which is not yet classified as a regeneration-ready. If tree stand will never grow to the size of saw timber due to e.g. tree species or unproductiveness of a habitat, the development class is defined according to forest age. In this case, the age defined on a breast-high is at minimum 25 years."* (Translated by authors from Äijälä et al. 2014.) The definition of mature stand is following: *"A forest is mature when a forest owner receives more economic gain by regenerating it than allowing it to grow."* (Translated by authors from Äijälä et al. 2014.)

We also investigated the average diameters of tree stands on logged experimental and control groups. They did not differ from each other (Table S1). Average diameters could not be calculated for unlogged stands as their data does not include diameters or any other features indicating the size of a tree stand.

Table S1. Average diameters of tree stands in logged spruce and pine mires with and without the candidate status.

Habitat type	Candidate status	Area (ha)*	Avg. diameter (cm)	SD
Spruce mires	With the status	86	18.11	4.91
	Without the status	39 264	18.69	5.16
Pine mires	With the status	493	17.12	4.49
	Without the status	69 480	17.73	5.95

*Areas differ from the ones used in the statistical analyses as stands lacking the average diameter were removed before calculating the average diameters for the total areas.

Appendix S3

Spatial data of the Finnish Forest Center

The Finnish Forest Center gathers and maintains open spatial data of forest resources and notifications of forest use. Forest resource data contains plenty of stand-based information of forests such as tree species, forest development class, habitat and soil type etc. (https://urly.fi/1jum). Grid-specific data with a resolution of 16 x 16 m is gathered via remote sensing utilizing laser scanning, aerial photographing, and measurements on experimental plots. Field inventories are used as a supportive method. Stand-specific information is calculated from sums and averages of grid cells. Data of each forest stand is updated on average every ten years as about 1.5 million hectares of forests are inventoried each year. Forest resource data includes majority of forest land of Finland, but especially state- and municipality-owned lands are not fully covered by it.

Data of notifications of forest use is composed of notifications submitted by a forest owner or more commonly, an agency that buys timber or executes loggings. It contains information about the planned logging, such as the specific area and purpose of the logging (https://urly.fi/1mtM). The notification is obligatory to fill and submit before any logging, with the exception of small-scale loggings such as household loggings for firewood or loggings of forests where the average diameter of tree stand is at maximum 13 cm.

Appendix S4

Size distributions of forest stand fragments

To delete most of the artificial forest stand fragments caused by data assembling, we analyzed size distributions of the fragments ≤ 1 ha in each data. Stands 0–0.02 ha were overrepresented and likely artificial (Fig. S4 and S5). However, deleting just them would likely have left many artificial stands in

the data as an average size of a forest stand in Finland is 1.2 ha (https://urly.fi/1juo). Since there were not a simple rule to decide the limit for the deletion, we estimated that deleting all the stands ≤ 0.14 ha would decrease the number of artificial stands, but save most of the real small stands such as woodland key habitats which median size is 0.35 ha (Kotiaho and Selonen 2006). Likely, not all artificial stands were removed, but most of the real stands were included to the data.

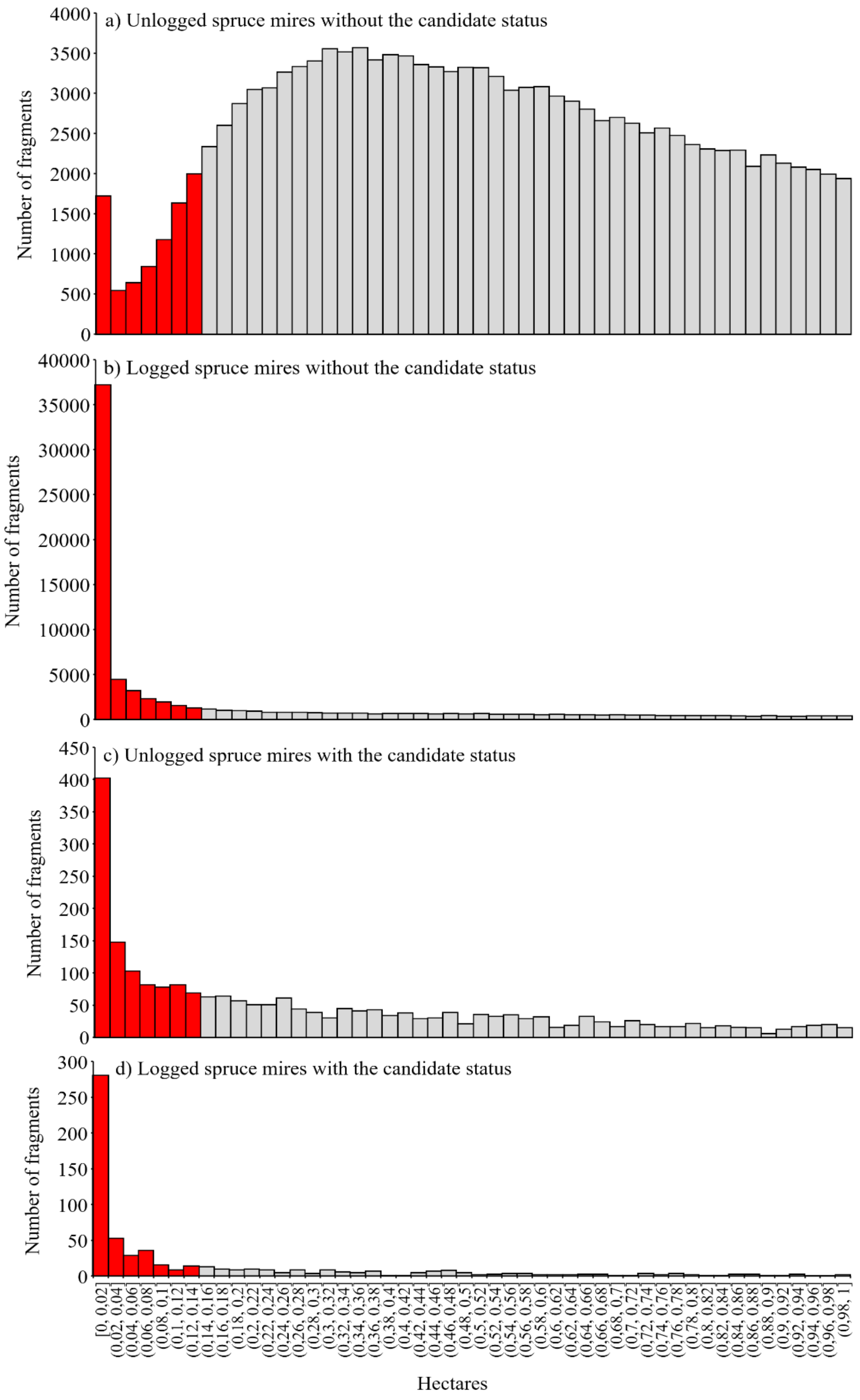

Figure S4. Size distributions of forest stand fragments on spruce mires. Only fragments ≤ 1 ha are shown. Stands ≤ 0.14 ha (marked as red) were deleted from the data. a) Unlogged spruce mires and

b) logged spruce mires without the candidate status. c) Unlogged spruce mires and d) logged spruce mires with the status.

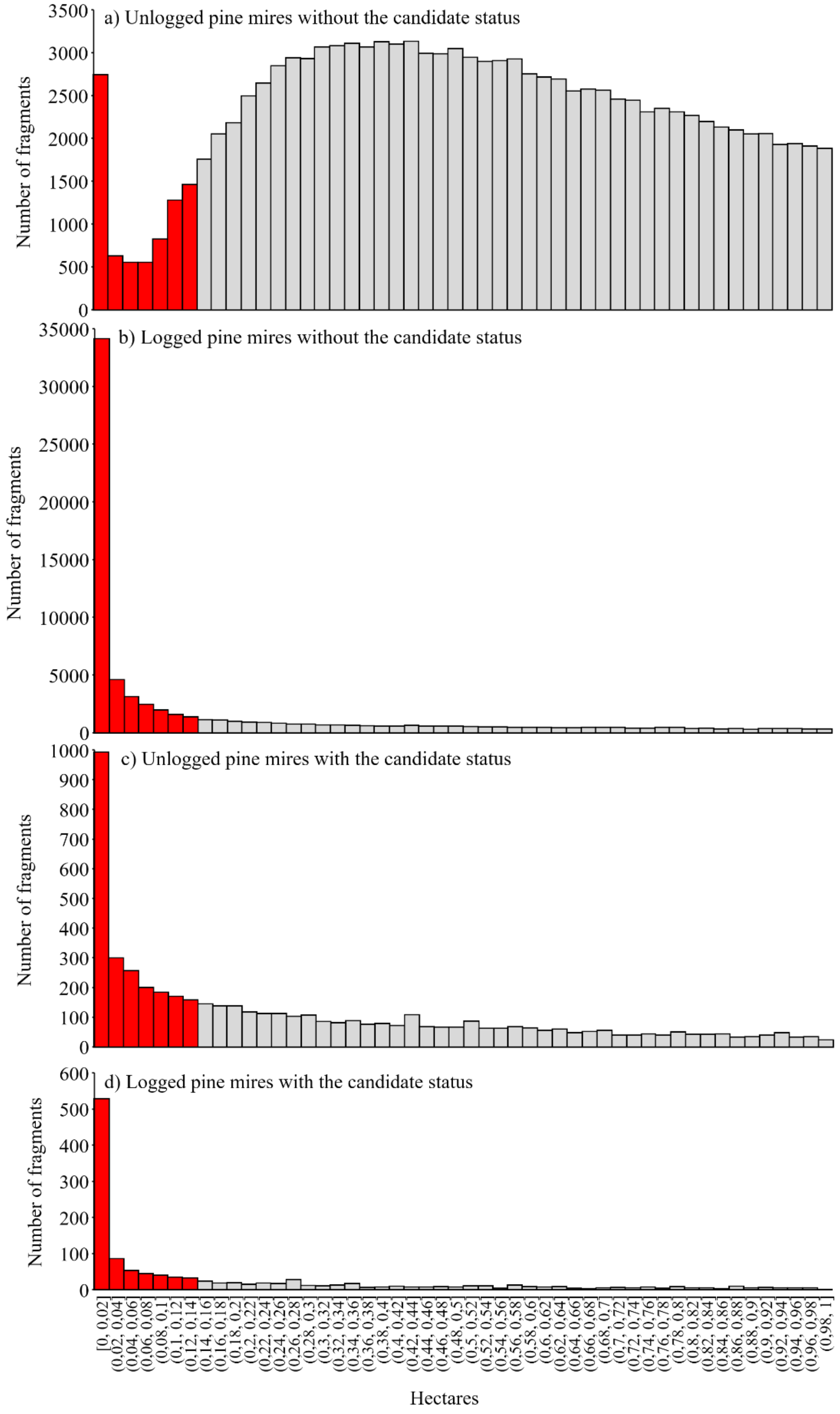

Figure S5. Size distributions of forest stand fragments on pine mires. Only fragments ≤ 1 ha are shown. Stands ≤ 0.14 ha (marked as red) were deleted from the data. a) Unlogged pine mires and b) logged pine mires without the candidate status. c) Unlogged pine mires and d) logged pine mires with the status.

Appendix S5

R-script for statistical analyses

```
n.habitat_name <- length(habitat_name[,1])

set.seed(5)

results.habitat_type.candidate <- rep(0, 1000)
results.habitat_type.not.candidate <- rep(0, 1000)

for(i in 1:1000) {
  ind <- sample.int(n.habitat_type)
  habitat_type.sample <- habitat_type$TREATMENT[ind]
  candidate.habitat_type.sample <- habitat_type.sample[habitat_type$TYPE ==
"CANDIDATE"]
  not.candidate.habitat_type.sample <- habitat_type.sample[habitat_type$TYPE ! =
"CANDIDATE"]
  habitat_type.candidate.prop <- sum(candidate.habitat_type.sample) /
length(candidate.habitat_type.sample)
  habitat_type.not.candidate.prop <- sum(not.candidate.habitat_type.sample) /
length(not.candidate.habitat_type.sample)
  results.habitat_type.candidate[i] <- habitat_type.candidate.prop
  results.habitat_type.not.candidate[i] <- habitat_type.not.candidate.prop
}
hist(results.habitat_type.candidate, family = "A", main = "",
xlab = "Harvesting rate (%)", ylab = "Frequency", col = "grey", ylim = c(0,120),
xlim = c(0.06,0.22), axis(1, at = seq (0.06, 0.22, by=0.002)), cex.lab = 1.3,
cex.axis = 1.3, cex.main = 1.3, cex.sub = 1.3)
```

```
sort(results.habitat_type.candidate,decreasing=F)[1:50]
```

References

Kotiaho, J.S., and V. Selonen. 2006. Analysis of the Quality and Reliability of the Survey on Habitats of Special Importance Defined in the Forest Act. The Finnish Environment 29/2006 (in Finnish, English abstract). http://hdl.handle.net/10138/38802

Äijälä, O., A. Koistinen, J. Sved, K. Vanhatalo, and P. Väisänen. (Ed.) 2014. Guidelines for forestry. Appendix 2.1 Forest development classes. Metsätalouden kehittämiskeskus Tapion julkaisuja (in Finnish). Retrieved 15 January 2020 from https://urly.fi/1plr.
